# Генетические маркеры о распространении древних морских охотников в Приохотье

**DOI:** 10.18699/VJ20.646

**Published:** 2020-08

**Authors:** B.A. Malyarchuk

**Affiliations:** Institute of Biological Problems of the North of the Far-East Branch of the Russian Academy of Sciences, Magadan, Russia

**Keywords:** mitochondrial DNA, CPT1A gene, human populations, paleogenomics, culture of marine hunters, Okhotsk Sea region, митохондриальная ДНК, ген CPT1A, популяции человека, палеогеномика, культура морских охотников, Охотоморский регион

## Abstract

Представлен обзор сведений о генетическом полиморфизме современного и древнего населения Севера Азии и Америки с целью реконструкции истории миграций древних морских охотников в Охотоморском регионе. Проанализированы данные о полиморфизме митохондриальной ДНК и распространенности «арктиче- ской» мутации – варианта rs80356779-A гена CPT1A. Известно, что «арктический» вариант гена CPT1A с высокой частотой распространен в современных популяциях эскимосов, чукчей, коряков и других народов Охотоморско- го региона, хозяйственный уклад которых связан с морским зверобойным промыслом. Согласно палеогеномным данным, самые ранние находки «арктического» варианта гена CPT1A обнаружены у гренландских и канадских па- леоэскимосов (4 тыс. лет назад), представителей токаревской культуры Северного Приохотья (3 тыс. лет назад) и носителей культуры позднего дзёмона острова Хоккайдо (3.5–3.8 тыс. лет назад). Результаты анализа позволили выявить несколько миграционных событий, связанных с распространением морских охотников в Охотоморском регионе. Самая поздняя миграция, оставившая следы у носителей культуры эпи-дзёмон (2.0–2.5 тыс. лет назад), привнесла с севера Приохотья на Хоккайдо и соседние территории Приамурья митохондриальную гаплогруппу G1b и «арктический» вариант гена CPT1A. Следы более ранней миграции, также привнесшей «арктическую» мута- цию, зарегистрированы у населения позднего дзёмона Хоккайдо (3.5–3.8 тыс. лет назад). Проведен филогенети- ческий анализ митохондриальных геномов, относящихся к редкой гаплогруппе C1a, встречающейся у населения Дальнего Востока и Японии, но в филогенетическом отношении родственной C1-гаплогруппам американских индейцев. Результаты показали, что дивергенция митохондриальных линий в пределах гаплогруппы C1a проис- ходила в диапазоне от 7.9 до 6.6 тыс. лет назад, а возраст японской ветви гаплогруппы C1a составляет ~5.2 тыс. лет. Пока неизвестно, связана ли эта миграция с распространением «арктического» варианта гена CPT1A или же присутствие C1a-гаплотипов у населения островов Японии маркирует собой еще один, более ранний, эпизод миграционной истории, связывающей население северо-западной Пацифики и Северной Америки.

## Abstract (en)

This is a review of studies on the genetic polymorphism of modern and ancient populations of the north of Asia and America, with the aim of reconstructing the history of migrations of ancient marine hunters in the Okhotsk Sea region. The data on mitochondrial DNA polymorphism and the “Arctic” mutation distribution – the rs80356779-A variant of the CPT1A gene – were analyzed. It is known that the “Arctic” variant of the CPT1A gene is widely distributed in modern populations of the Eskimos, Chukchis, Koryaks, and other peoples of the Okhotsk Sea region, whose economic structure is associated with marine hunting. According to paleogenomic data, the earliest cases of the “Arctic” variant of the CPT1A gene were found in the Greenland and Canadian Paleoeskimos (4 thousand years ago), among representatives of the Tokarev culture of the Northern Priokhotye (3 thousand years ago), and among the bearers of the culture of the late Jomon of Hokkaido (3.5–3.8 thousand years ago). The results of the analysis revealed several migration events associated with the spread of marine hunters in the Okhotsk Sea region. The latest migration, which left traces on bearers of the Epi-Jomon culture (2.0–2.5 thousand years ago), introduced the mitochondrial haplogroup G1b and the “Arctic” variant of the CPT1A gene from the north of Priokhotye to Hokkaido and neighboring territories of the Amur Region. Traces of earlier migration, which also brought the “Arctic” mutation, were recorded in the Hokkaido population of the late Jomon period (3.5–3.8 thousand years ago). A phylogenetic analysis of mitochondrial genomes belonging to the rare haplogroup C1a, found in populations of the Far East and Japan, but phylogenetically related to the C1-haplogroups of the Amerindians, was carried out. The results of the analysis showed that the divergence of mitochondrial lineages within the C1a haplogroup occurred in the range from 7.9 to 6.6 thousand years ago, and the age of the Japanese branch of the C1a haplogroup is approximately 5.2 thousand years. It is not yet known whether this migration is associated with the spread of the “Arctic” variant of the CPT1A gene or the presence of C1a haplotypes in the population of the Japanese islands marks another, earlier, episode of the migration history linking the populations of Northwest Pacific and North America.

## Введение

Согласно результатам археологических и палеогеномных исследований, 4.5–2.8 тысячелетия назад в обширном регионе – от Чукотки и Аляски и до Гренландии – суще- ствовала общность племен (палеоэскимосов), объеди- нявшая искусных охотников на морских млекопитающих (Rasmussen et al., 2010; Flegontov et al., 2019). Предпо- лагается, что формирование высокоспециализированной культуры морских зверобоев проходило главным обра- зом на американском континенте и связано с развитием «арктической традиции малых орудий», а на Чукотке палеоэскимосские памятники возрастом от 3.5 до 2.9 тыс. лет обнаружены в ограниченном числе мест – на острове Врангеля и поселении Уненен (Гребенюк и др., 2019). По палеогеномным данным, первая миграция предков палео- эскимосов из Сибири в Америку произошла ~ 5.5 тыс. лет назад (Rasmussen et al., 2010). В генетическом отношении палеоэскимосы ближе всего к современным чукотско-кам- чатским народам (корякам, чукчам, ительменам), а не аме- риканским индейцам (Flegontov et al., 2019). Результатом другой миграции из Сибири в Америку (~ 4 тыс. лет назад) стало появление собственно современных эскимосов (неоэскимосов) севера Америки, Гренландии и Чукотки (Achilli et al., 2013). Неоэскимосы формировались на па- леоэскимосской основе, но, несмотря на объединяющую носителей этих культур традицию морского зверобойного промысла, в генетическом отношении они различались (Rasmussen et al., 2010; Raghavan et al., 2014; Flegontov et al., 2019; Sikora et al., 2019).

Установлены также две волны обратных миграций из Америки на крайний Северо-Восток Азии. Одна из них связана с миграцией палеоэско-алеутских групп ~3.5 тыс. лет назад, а другая – с миграциями неоэскимосов ~2.5 тыс. лет назад (Гребенюк и др., 2019; Flegontov et al., 2019; Grugni et al., 2019; Sikora et al., 2019). В результате первой из указанных обратных миграций на Чукотке возникла палеоэскимосская традиция, а в Северном При- охотье – токаревская культура (Гребенюк и др., 2019). Результатом второй обратной миграции стало развитие неоэскимосских культур в Берингоморье (Raghavan et al., 2014; Flegontov et al., 2019).

## Генетические маркеры древних морских охотников

Генетическая реконструкция событий, произошедших на крайнем Севере Азии и Америки, стала возможной благодаря исследованиям распределения вариантов митохондриальной ДНК (мтДНК) и Y-хромосомы, наследуемых по материнской и отцовской линиям соответственно, у современного и древнего населения этого региона. Исследования полиморфизма мтДНК показали, что только несколько митохондриальных гаплогрупп объединяют генетически популяции крайнего Севера Америки (эскимосов) и Северо-Востока Азии (эскимосов, чукчей и коряков) – это гаплогруппы A2a, A2b, D2a и D4b1a2a1a (Derenko et al., 2007; Tamm et al., 2007; Dryomov et al., 2015). Гаплогруппа D2a маркирует собой предков палеоэскимосов, поскольку она характерна для пред- ставителей палеоэскимосских культур Саккак и Дорсет, а также токаревской культуры Северного Приохотья, а остальные гаплогруппы связаны уже с относительно не- давней неоэскимосской экспансией (Raghavan et al., 2014; Flegontov et al., 2019; Sikora et al., 2019). Аналогично по распределению вариантов Y-хромосомы только гапло- группа Q-B143 маркирует древний палеоэско-алеутский компонент, отмеченный также у современных коряков, чукчей и юкагиров (Rasmussen et al., 2010; Malyarchuk et al., 2011; Karmin et al., 2015; Grugni et al., 2019). Между тем присутствие мужской гаплогруппы Q-B34 у азиатских эскимосов и коряков связано с обратной миграцией, при- ведшей к появлению неоэскимосских культур на Чукотке (Grugni et al., 2019).

Из аутосомных генетических вариантов наиболее инте- ресна «арктическая» мутация гена CPT1A, кодирующего карнитин пальмитоилтрансферазу типа IA – один из клю- чевых ферментов транспорта жирных кислот в митохон- дрии. В результате нуклеотидной замены G→A в локусе rs80356779 гена CPT1A образуется аминокислотная за- мена пролина на лейцин в позиции 479 фермента CPT1A (замена Р479L), которая относится к числу патологиче- ских, поскольку приводит к понижению ферментативной активности CPT1A (Greenberg et al., 2009). Проведенные исследования установили, что «арктическая» мутация rs80356779-A с высокой частотой распространена только на крайнем Севере Азии и Америки (Rajakumar et al., 2009; Lemas et al., 2012; Clemente et al., 2014; Малярчук и др., 2016). Ее частота составила более 70 % у американских и гренландских эскимосов, 66 – у коряков, 56 – у чукчей и 30 % – у охотских эвенов (Малярчук и др., 2016). Резуль- таты филогенетического анализа протяженных участков гена CPT1A продемонстрировали однократное возник- новение мутации rs80356779-A у эскимосов, чукчей и коряков (Clemente et al., 2014), а ее появление у эвенов и эвенков связано не с независимым происхождением этого варианта полиморфизма, а с межэтническими контактами (Малярчук и др., 2016).

Обнаружено, что поддержанию высокой частоты «арктического» варианта гена CPT1A у эскимосов, чукчей и коряков способствовал отбор, связанный, в первую очередь, с адаптацией к традиционной диете морских зверо- боев, основанной на потреблении жира и мяса ластоногих и китов (Clemente et al., 2014). По всей видимости, в ответ на высокий уровень кетогенеза, возникающий при посто- янном потреблении жирной пищи, у морских охотников произошли некоторые изменения метаболизма, например у них отпала необходимость в высокой активности фер- ментов метаболизма полиненасыщенных жирных кислот, которыми богаты продукты морского зверобойного про- мысла. Аминокислотная замена Р479L как раз приво- дит к снижению каталитической активности фермента CPT1A (Greenberg et al., 2009). В результате носители «арктического» варианта в большей степени защищены от кетогенеза, что вполне оправданно при соблюдении традиционной диеты, но в современных условиях (в слу- чае отхода от традиционной диеты) эта аминокислотная замена стала вредной. Так, у эскимосов Северной Аме- рики и Гренландии – носителей «арктической» мутации в гомозиготном состоянии (их частота составляет 40–70 % в различных популяциях) – дефицит фермента CPT1A со- провождается гипокетонной гипогликемией, синдромом внезапной детской смерти, бóльшей предрасположенно- стью к ожирению, диабету 2-го типа, жировой болезни печени и др. (Greenberg et al., 2009). Вызывает тревогу, таким образом, высокая частота гомозиготного носитель- ства «арктического» варианта» (генотип rs80356779-AA) у коренного населения крайнего Северо-Востока Азии (47 % у коряков, 33 у чукчей и 8 % у охотских эвенов), тем более в условиях отсутствия неонатального скри- нинга «арктической» мутации у новорожденных детей коренного населения.

## Распространенность «арктического» варианта гена CPT1A в современных и древних популяциях

Предполагается, что распространению «арктического» варианта гена CPT1A в популяциях коренного населения Северо-Восточной Азии способствовали миграции мор- ских охотников вдоль побережий северных морей (Ма- лярчук, 2018). Кроме эскимосов, чукчей и коряков, случаи этой мутации мозаично были зарегистрированы у эвенков Якутии (с частотой 1 %) (Малярчук и др., 2016), долгано- нганасанского населения Таймыра (3 %) (Smolnikova et al., 2015), нанайцев северокитайской провинции Хэйлунцзян (10.3 %) (Li et al., 2018). Поскольку в соседних по от- ношению к указанным этническим группам популяциях «арктический» вариант гена CPT1A отсутствовал, то наиболее вероятным представляется, что случаи нахождения варианта rs80356779-A вдали от Чукотки и Северного Приохотья могут объясняться миграциями древних морских охотников. Согласно археологическим данным, груп- пы морских охотников из Берингоморья проникали на Таймыр ~ 3 тыс. лет назад (Гурвич, Симченко, 1980). Неоднократно также археологами отмечалась экспансия морских охотников с севера на юг вдоль побережья Охот- ского моря (Лебединцев, 1990; Befu, Chard, 2017).

Результаты молекулярного датирования показали, что эволюционный возраст «арктического» варианта гена CPT1A составляет от 6 до 23 тыс. лет (Clemente et al., 2014). Между тем сведения палеогеномных исследований позволяют считать, что «арктическая» мутация появилась у коренного населения Арктического и Охотоморского регионов на протяжении последних четырех тысячелетий. Согласно палеогеномным данным, «арктическая» мута- ция отсутствовала у представителей позднего палеолита арктической Восточной Сибири (стоянка Яна, 32.5 тыс. лет назад) (Sikora et al., 2019) и Юго-Восточной Сибири (стоянка Мальта, 24 тыс. лет назад) (Raghavan et al., 2014), а также в Северной Америке у представителя культуры Кловис (12.6 тыс. лет назад) (Rasmussen et al., 2014) – де- вочки из погребения Upward Sun River (Аляска, 11.5 тыс. лет назад) (Moreno-Mayar et al., 2018) и Кенневикского человека (8.3–9.2 тыс. лет назад) (Rasmussen et al., 2015). В мезолите и неолите «арктическая» мутация также не была выявлена по результатам исследований древнего индивидуума из Дуванного Яра (Чукотка, ~ 9.8 тыс. лет назад), а также древних жителей пещеры Чертовы ворота (Приморье, ~ 7.5 тыс. лет назад) (Sikora et al., 2019). «Арктический » вариант отсутствовал также у индивидуумов со стоянки Усть-Белая (Прибайкалье, 4.5–6.6 тыс. лет назад) (Sikora et al., 2019).

Впервые «арктический» вариант гена CPT1A был об- наружен в гетерозиготном состоянии у палеоэскимоса, представляющего культуру Саккак из Гренландии (4 тыс. лет назад), а также с частотой ~ 50 % у канадских и грен- ландских представителей палеоэскимосской культуры Дорсет (1.4–1.6 тыс. лет назад) (Rasmussen et al., 2010; Clemente et al., 2014). «Арктический» вариант зарегистри- рован также у двух представителей токаревской культуры (Северное Приохотье, 3 тыс. лет назад) и у древних жите- лей эскимосского поселения Эквен (Чукотка, 1.9–2.1 тыс. лет назад) (Sikora et al., 2019). Недавние исследования продемонстрировали, что и для представителей Южно- го Приохотья – носителей культуры позднего дзёмона (Хоккайдо, 3.5–3.8 тыс. лет назад) также был характерен «арктический» вариант гена CPT1A (Kanzawa-Kiriyama et al., 2019). Авторы предположили, что частота варианта rs80356779-A в популяции позднего дзёмона Хоккайдо была высокой, а ее закреплению способствовал тот факт, что древние жители активно охотились на морских животных (морских котиков, сивучей, морских львов, дельфинов). Возможно, что присутствие «арктического» варианта у представителей позднего дзёмона Хоккайдо связано с воздействием древних северо-восточноазиат- ских популяций, повлиявших также и на предков других народов Приамурья и Сахалина – например нивхов, у которых также обнаружен «арктический» вариант гена CPT1A (Zhou et al., 2019). Между тем результаты исследо- вания полиморфизма мтДНК показали, что для древнего и современного населения Хоккайдо преемственность от древности до современности сохраняется для мито- хондриальных гаплогрупп N9b, D4h2 и M7a, которые отсутствуют у народов Северо-Востока Азии (коряков, ительменов, чукчей) (Adachi et al., 2018). Общим же зве- ном для всех указанных популяций служит присутствие гаплогруппы G1b. Эта гаплогруппа мтДНК появилась у представителей эпи-дзёмона на Хоккайдо 2.0–2.5 тыс. лет назад и распространена у современных айнов с частотой около 16 % (Adachi et al., 2011). Ее появление явно связано с миграциями с северо-востока, поскольку гаплогруппа G1b установлена как у мезолитического индивидуума из Дуванного Яра (Чукотка, 9.8 тыс. лет назад), так и у токаревцев (3 тыс. лет назад) (Sikora et al., 2019). По всей видимости, на Хоккайдо эта гаплогруппа мтДНК попала в связи с распространением токаревской культуры на юг Приохотья.

Экспансия носителей митохондриальной гаплогруппы G1b на рубеже нашей эры в популяциях Охотоморского региона, вероятно, повлекла за собой и распространение «арктического» варианта гена CPT1A. Однако поскольку гаплогруппа G1b не обнаружена на Хоккайдо в более раннее время (т. е. 3.5–3.8 тыс. лет назад), то вполне возможно, что источник «арктического» варианта гена CPT1A у них мог быть связан с другой, более ранней, миграцией. Анализ данных о полиморфизме мтДНК у на- селения северной части Восточной Азии показывает, что в литературе имеются сведения о присутствии в восточ- ноазиатских популяциях очень редкой митохондриальной гаплогруппы C1a, которая в филогенетическом cмысле является сестринской ветвью по отношению к распро- страненным у индейцев Америки гаплогруппам C1b, C1c и C1d. Гаплогруппа C1a обнаружена у японцев (0.5 %) (Maruyama et al., 2003), ульчей (0.6 %) (Starikovskaya et al., 2005), ороков Сахалина (11.5 %) (Бермишева и др., 2005), нанайцев (1.2 %) (Tamm et al., 2007), дауров (2.2 %) (Kong et al., 2003), монголов (1.3 %) (Kolman et al., 1996; Derenko et al., 2007), алтайцев (0.7 %) (Dulik et al., 2012), бурят (0.7 %) (Derenko et al., 2007), киргизов (0.5 %) (Tamm et al., 2007), казахов (0.8 %) (Tamm et al., 2007). Предполагается, что присутствие гаплогруппы C1a на северо-востоке Азии связано с обратной миграцией из Америки (Tamm et al., 2007), произошедшей ~ 8.6 тыс. лет назад, судя по эволю- ционному возрасту этой гаплогруппы мтДНК (Derenko et al., 2010). Конечно, по результатам филогенетического анализа и возрасту гаплогруппы трудно определить, ког- да именно была предполагаемая миграция. Неизвестно, появились ли мутации, определяющие гаплогруппу C1a (рисунок), в Азии или же произошел перенос в Азию уже сформировавшейся гаплогруппы C1a, возникшей очень локально в Америке.

**Fig. 1.**
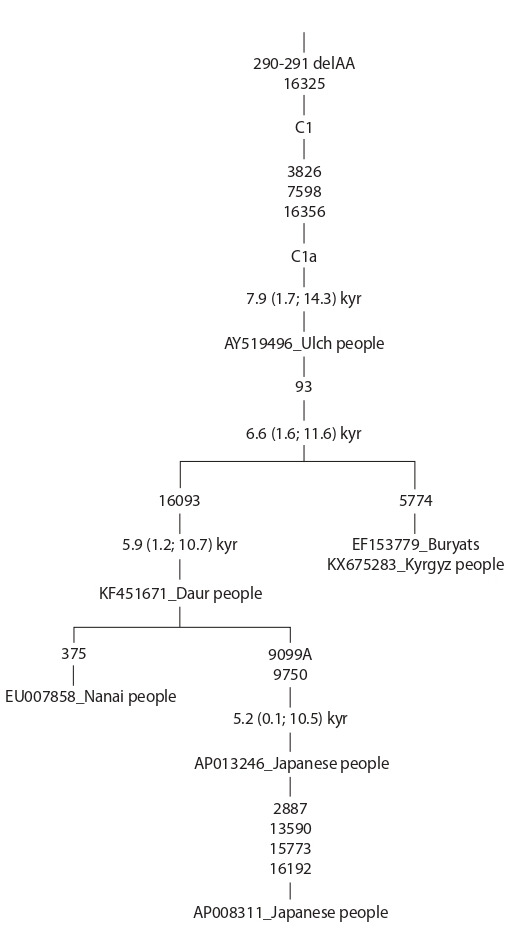
The phylogenetic tree of whole mitochondrial genomes belonging to haplogroup C1a. Transitions are indicated on tree branches; for transversions, the results of nucleotide replacements are shown; deletions are marked as del. The evolutionary ages of mtDNA clusters (in thousands of years, kyr) are given in accordance with the mutation rate in the whole mitogenome, equaling 1.665 × 10^–8^ substitutions per site per year (Soares et al., 2009). For mitogenomes, GenBank numbers and ethnicities are indicated. The phylogenetic tree is built with the program mtPhyl 4.015 (https://sites.google.com/site/mtphyl/home).

Филогенетический анализ всех известных к настоящему времени C1a-митогеномов показывает, что эволюционный возраст гаплогруппы составляет ~8 тыс. лет, а дивергенция основных гаплотипов от корневой последовательности мтДНК произошла ~6.6 тыс. лет назад, при этом япон- ская ветвь гаплогруппы C1a имеет возраст ~5.2 тыс. лет (см. рисунок). Таким образом, учитывая достаточно ши- рокий диапазон времени формирования «арктического» варианта гена CPT1A (от 6 до 23 тыс. лет назад), можно предположить, что C1a-миграция из американской части Берингии могла быть осуществлена носителями «аркти- ческого» варианта гена CPT1A. В таком случае находит объяснение присутствие как C1a-гаплотипов мтДНК у современных японцев и народов Сахалина и Приамурья, так и «арктического» варианта гена CPT1A у предста- вителей позднего дзёмона Хоккайдо и современных на- родов Сахалина и Приамурья. В настоящее время ареал C1-гаплогрупп у народов Америки смещен к югу, однако палеогеномные исследования установили присутствие C1b-гаплотипа на Аляске 11.5 тыс. лет назад (Tackney et al., 2015). Поэтому вполне вероятно, что митохондри- альные генофонды берингийцев включали в свой состав C1-линии, одна из которых могла стать родоначальницей гаплогруппы C1a.

В отношении происхождения «арктического» варианта гена CPT1A установлено, что для варианта rs80356779-A предковым является гаплотип, характерный для населения Восточной Азии (Clemente et al., 2014). Однако этапы дальнейших изменений этого гаплотипа, приведших к возникновению «арктической» мутации, равно как и место

## Заключение

Полученные результаты позволили выявить несколько миграционных событий, связанных с распространением морских зверобоев в Охотоморском регионе. Самая позд- няя миграция, оставившая следы у носителей культуры эпи-дзёмон, привнесла с севера Приохотья на Хоккайдо и соседние территории Приамурья митохондриальную гаплогруппу G1b и «арктический» вариант гена CPT1A. Следы более ранней миграции, также привнесшей «арктическую » мутацию, зарегистрированы у населения позднего дзёмона Хоккайдо. Пока неизвестно, связана ли эта миграция с распространением митохондриальной линии C1a или же присутствие C1a у населения островов Японии маркирует собой еще один, более ранний, эпизод ми- грационной истории, связывающей население Берингии и всего Охотоморского региона. Следует отметить, что в этнологической литературе уже давно существует пред- положение об этногенетическом родстве предков нивхов, чукчей, коряков, эскимосов и американских индейцев (Jochelson, 1926). Предполагается также существование в прошлом циркумохотской культурной общности, имевшей глубокие связи с сопредельными культурами азиатского побережья и островов северо-западной Пацифики и Се- верной Америки (Лебединцев, 2003).

## Conflict of interest

The authors declare no conflict of interest.
